# Clinical Profile and Outcomes of Nephrotic Syndrome With Acute Kidney Injury in Adults: A Prospective Cohort Study

**DOI:** 10.7759/cureus.47626

**Published:** 2023-10-25

**Authors:** Rajaram Yadav, Om Kumar, Amresh Krishna, Prit P Singh, Prem S Patel

**Affiliations:** 1 Nephrology, Indira Gandhi Institute of Medical Sciences, Patna, IND

**Keywords:** acute kidney injury, nephrotic syndrome, aki, renal recovery, akin staging, renal dysfunction, adults

## Abstract

Background: Nephrotic syndrome (NS) is characterized by various clinicopathological conditions like proteinuria, hypoalbuminemia, and anasarca. Patients with NS are prone to experience associated problems like acute kidney injury (AKI). The present study aimed to investigate the clinical profile and outcomes of NS with AKI in adults.

Materials and methods: This prospective, observational study was conducted over a period of one year. Adult patients with NS diagnosed with AKI were enrolled in the study. Data were collected at baseline and patients were followed up for at least three months.

Results: A total of 60 patients were enrolled. The majority of the patients (78.3%) were aged between 18 and 30 years. Anemia was observed among 96.7% of the patients. A significant improvement was observed in the mean levels of proteinuria (5.80 vs. 1.70 gm/dL; P < 0.001), total cholesterol (270.00 mg/dL vs. 160.00 mg/dL), serum creatinine (2.18 mg/dL vs. 1.68 mg/dL; P < 0.001), and serum albumin (1.86 gm/dL vs. 3.29 gm/dL; P < 0.001) at baseline to three months. Pre-renal AKI was diagnosed in 95% of patients. According to histological classification, 19 patients had minimal change disease, whereas focal segmental glomerulosclerosis was observed in 23 patients. It was observed that 96.7% of the patients did not necessitate the need for renal replacement therapy.

Conclusion: The present study successfully examined the clinical profile and outcomes of adults with NS and AKI. The findings provide valuable insights into the characteristics and prognosis of this patient population, contributing to a better understanding of NS with AKI in adults.

## Introduction

Nephrotic syndrome (NS) is a debilitating condition characterized by significant proteinuria, hypoalbuminemia, edema, and dyslipidemia. It primarily affects adults and can be associated with various renal pathologies. However, the presence of acute kidney injury (AKI) in conjunction with NS poses additional challenges and potentially worsens patient outcomes.

Despite advancements in renal replacement therapy (RRT), AKI still carries a high risk of unfavorable outcomes. AKI is characterized by a sudden decline in kidney function, leading to impaired waste excretion, reduced urine output, and typically developing rapidly within hours to days. It frequently occurs among hospitalized individuals, particularly those who are critically ill [1].

The causes and treatment approaches for AKI vary significantly not only between developing and developed countries but also within the same country, varying from one medical facility to another. Several factors, such as location, level of expertise, and available resources at each center, influence these differences. Additionally, similar to adults, AKI poses a substantial risk for the subsequent development of chronic kidney disease (CKD) in children who survive the initial episode. The incidence of AKI is observed in approximately 10-15% of hospitalized patients, whereas more than 50% of patients admitted from intensive care units report an incidence of AKI.

Approximately 20% of patients, particularly those who are critically ill, may experience AKI as a result of commonly prescribed medications such as aminoglycosides, amphotericin, non-steroidal anti-inflammatory drugs, methotrexate, cisplatin, cyclosporine, angiotensin-converting-enzyme inhibitors, and angiotensin-receptor blockers (ARBs). These drugs have been identified as potential risk factors for causing AKI [1].

There is a general consensus that the severity and duration of an AKI episode have a significant impact on long-term outcomes, such as survival and kidney recovery. Patients with AKI who experience a temporary decline in kidney function may fully recover without permanent loss of nephrons. However, AKI cases involving substantial damage to nephrons can lead to the development of post-AKI CKD and potentially progress to end-stage renal disease (ESRD) [2]. The recovery of AKI occurs in almost all patients with mild to moderate degree of renal dysfunction; however, a few cases of NS with severe AKI may not recover and progress to CKD, finally leading to ESRD. Therefore, timely detection and diagnosis of AKI are crucial for accurate prognosis and appropriate management [3]. To achieve better patient outcomes, there is a need for deeper study. Thus, the present study aimed to investigate the clinical profile and outcomes of NS with AKI in adults.

## Materials and methods

Study design

This was a prospective, observational study conducted over a period of one year. The study was approved by the Indira Gandhi Institute of Medical Sciences, Patna (approval: 275/IEC/IGIMS/2021) and was performed in accordance with the Declaration of Helsinki and the International Conference on Harmonization guidelines. Written informed consent was obtained from all the participants prior to enrollment in this study.

Study participants

Adult patients of either gender with age ≥ 18 years to 70 years who were diagnosed with NS and AKI were included in the study.

Patients diagnosed with diabetes mellitus, CKD, systemic illness causing proteinuria, and patients with acute nephritic syndrome & rapidly progressive glomerular nephritis were excluded from the study.

Data collection

Data were recorded for patients who met the eligibility criteria, including demographic, clinical, and laboratory parameters (systolic and diastolic blood pressure along with findings of renal histology). Systemic diseases were ruled out through a detailed history, clinical examination, and autoimmune antibody testing, including antinuclear antibody (ANA) by immunofluorescence assay (IFA), anti-double-stranded DNA (anti-dsDNA) antibody, anti-Smith (anti-Sm) antibody, proteinase 3-antineutrophil cytoplasmic antibody (PR3-ANCA), and myeloperoxidase-antineutrophil cytoplasmic antibody (MPO-ANCA). The enzyme-linked immunosorbent assay (ELISA) method was used to detect and quantify these antibodies. The need for RRT was also documented.

Definitions

An NS was defined as the presence of edema, proteinuria in excess of 3.5 g/day, and serum albumin less than 30 g/L.

Stages of AKI

Stage 1 AKI was defined as 1.5-1.9 times baseline or ≥0.3 mg/dl increase in serum creatinine and urine output <0.5 ml/kg/hour for six to 12 hours.

Stage 2 AKI was defined as 2-2.9 times baseline in serum creatinine or urine output <0.5 ml/kg/hour for ≥12 hours.

Stage 3 AKI was defined as an increase in serum creatinine to three times baseline or ≥4 mg/dL or the initiation of RRT or in patients aged <18 years, decrease in eGFR to <35 ml/min/1.73m2 or urine output <0.3 ml/kg/hour for ≥24 hours or anuria for ≥12 hours [4].

Follow up

After admission, patients were followed up for a minimum of three months. Upon clinical improvement, patients were discharged and subsequently followed up in the outpatient department. Monthly or biweekly biochemical investigations and urine protein measurements were conducted based on the patients’ clinical situation.

Statistical analysis

Data were analyzed using Statistical Package for the Social Sciences (SPSS) version 21 (IBM Corp., Armonk, NY). Descriptive statistics were used to describe categorical variables (frequency and percentages) and continuous variables (mean and standard deviation (SD)). A paired sample T-test was used to compare biochemical parameters of NS from baseline and three months. A P < 0.05 was considered statistically significant.

## Results

This study included a total of 60 patients, with 78.3% of them under the age of 30 years. The majority (56.7%) of the study population was male and 43.3% of the patients were females. It was observed that 96.7% of the patients were anemic. According to an immunological study, 5% of patients had low C3. Further, it was observed that 100% of the patients had normal C4 (Table 1).

**Table 1:** Demographic and baseline characteristics of patients Data are presented as n (%) unless otherwise specified.

Patient characteristics	Total (N = 60)
Age group (years)	
<30	47 (78.3)
30-60	11 (18.3)
>60	2 (3.3)
Gender	
Male	34 (56.7)
Female	26 (43.3)
Blood pressure (mmHg), mean (SD)	
Systolic	129 (15.6)
Diastolic	79.1 (5.6)
Presence of anemia	
Present	58 (96.7)
Absent	2 (3.3)
Immunological study	
C3	
Low	3 (5.0)
Normal	57 (95.0)

The biochemical parameters of the NS were compared between baseline and three months (Table 2). The mean levels of proteinuria (5.80 vs. 1.70 gm/day; P < 0.001), total cholesterol (270.00 mg/dL vs. 160.00 mg/dL), and serum creatinine (2.18 mg/dL vs. 1.68 mg/dL; P < 0.001) showed a significant decrease from baseline to three months. On the other hand, the mean serum albumin levels exhibited a significant increase from baseline to three months of follow-up (1.86 gm/dL vs. 3.29 gm/dL; P < 0.001).

**Table 2:** Comparison of biochemical parameters of nephrotic syndrome from baseline to three months Data are presented as mean (SD).

Parameter	Baseline	At 3 months	P-value
Proteinuria (gm/day)	5.80 (2.03)	1.70 (2.04)	<0.001
Serum albumin (gm/dL)	1.86 (0.43)	3.29 (0.86)	<0.001
Total cholesterol (mg/dL)	270 (43.00)	160 (62.00)	<0.001
Serum creatinine (mg/dL)	2.18 (0.53)	1.68 (1.38)	<0.001

Among the patients observed, 95% (n = 57) were diagnosed with pre-renal AKI, while 5% (n = 3) had renal AKI specifically caused by sepsis. All patients with pre-renal AKI exhibited hypoalbuminemia and were using diuretics, and 26.7% (n = 16) had a history of prolonged non-steroidal anti-inflammatory drugs (NSAIDs) use. The observations revealed that 32 patients had stage I Acute Kidney Injury Network (AKIN) classification, while 28 had stage II AKIN. Among the patients with stage I AKIN, 96.9% experienced recovery, while 3.1% progressed to CKD. Similarly, among those with stage II AKIN, 82.1% achieved recovery, while 17.9% progressed to CKD (Table 3).

**Table 3:** Etiology and outcomes of AKI Data are presented as n (%). AKI, acute kidney injury; AKIN, Acute Kidney Injury Network; CKD, chronic kidney disease; NSAIDs, non-steroidal anti-inflammatory drugs.

Class of AKI	N = 60
Pre-renal	57 (95.0)
Diuretics	60 (100.0)
NSAIDs	16 (26.7)
Hypoalbuminemia	60 (100.0)
Renal	3 (5.0)
Sepsis	3 (100.0)
AKI stage (AKIN)	
AKIN I	n = 32
Recovered	31 (96.9)
Progressed to CKD	1 (3.1)
AKIN II	n = 28
Recovered	23 (82.1)
Progressed to CKD	5 (17.9)

**Figure 1:**
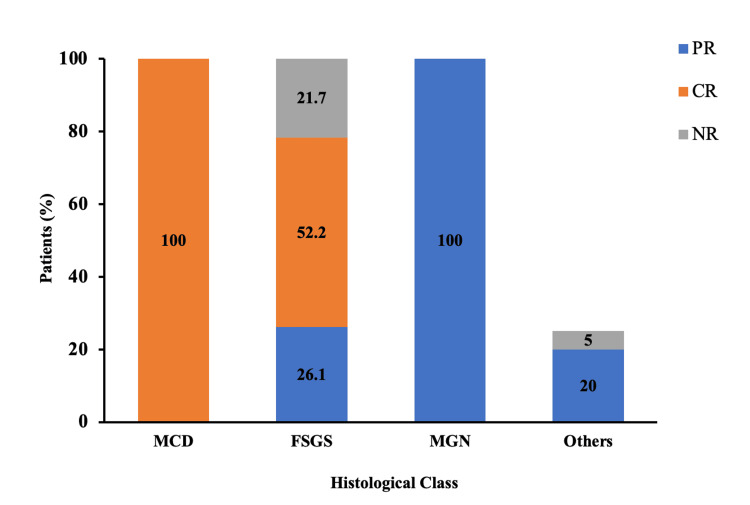
Distribution of patients according to histological class of study population Other causes of nephrotic syndrome include C3 glomerulonephritis and IgA nephropathy. FSGS, focal segmental glomerulosclerosis; MCD: minimal change disease; MGN, membranous glomerulonephritis; CR, complete response; NR, no response; PR, partial remission.

Among the histological classes, 19 patients were diagnosed with minimal change disease, and all (100%) exhibited a complete response (CR). Focal segmental glomerulosclerosis (FSGS) was observed in 23 patients, with 52.2% achieving CR, 26.1% experiencing partial remission (PR), and 21.7% showing no response (NR). Among the 13 patients with membranous glomerulonephritis, all of them demonstrated PR. The remaining causes of NS included C3 glomerulonephritis and IgA nephropathy (n = 5), with four patients showing NR and one achieving PR (Figure 1).

**Figure 2:**
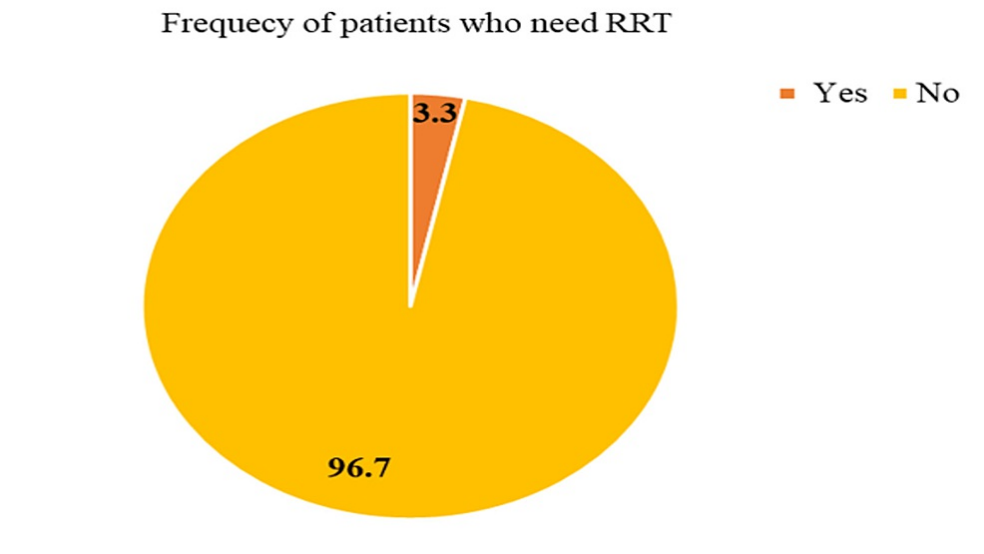
Need for renal replacement therapy (RRT) in the study population

It was observed that 96.7% of the patients did not require RRT, while 3.3% required RRT (Figure 2).

## Discussion

Patients with NS typically seek medical attention from clinicians due to generalized body swelling, i.e., anasarca. These patients are more susceptible to complications such as AKI, hypovolemia, sepsis, and thrombosis. Among these complications, AKI poses a frequent and concerning clinical challenge. However, the availability of data on the characteristics of adult patients with NS and AKI is limited. Therefore, this prospective study aimed to investigate the baseline characteristics and treatment outcomes of NS with AKI in our institution.

Initially, 200 patients with idiopathic NS were screened for AKI. Among them, 30% (n = 60) were found to have AKI. It was observed that most study population (78.3%) were below 30 years old. Furthermore, within the study population, 56.7% were male. A study by Yaseen et al. [5] also reported a male predominance, with 62.2% of the patients being male. In that study, the majority of patients (58.6%) fell within the age group of 18-40 years. Similarly, in a study conducted by Lin et al. [6], AKI was found in 29.7% of the 367 patients examined and 78.0% were male.

Remarkably, when analyzed using the World Health Organization criteria for anemia, it was observed that 96.7% of the patients with NS and AKI were found to be anemic. In comparison, only 3.3% had normal hemoglobin levels. Furthermore, among 119 patients of AKI with NS, a significant majority (69.7%) was found to be anemic [5]. However, despite these findings, the relationship between anemia and AKI, as well as the influence of anemia on long-term mortality in critically ill patients, remains unanswered.

During the immunological investigation, none of the patients tested positive for antinuclear antibodies, and their C4 levels were within the normal range. Only 5% of patients exhibited reduced C3 levels. In contrast, a study conducted by Aditya and colleagues [7] reported different findings. Among the total 64 patients in their study, 81% had low levels of C3, while 15.6% had low levels of C4. Furthermore, 15.6% (n = 10) of the patients exhibited low levels of both C3 and C4 [8]. These variations in the prevalence of reduced C3 and C4 levels between the two studies suggest potential differences in the underlying immunological mechanisms and disease characteristics among the patient populations. Further research is necessary to better understand the significance of these findings and their implications for the diagnosis and management of patients with NS and AKI.

After a period of three months, the mean proteinuria decreased from 5.80 g/day to 1.70 g/day. Additionally, the mean serum albumin levels, which were 1.86 g/dL at baseline increased to 3.29 g/dL after three months. This improvement in serum albumin levels corresponded to a decrease in proteinuria. Furthermore, at baseline, the mean total cholesterol was 270 mg/dL, which decreased to 160 mg/dL after three months. This reduction in total cholesterol level indicates a positive response to the treatment.

Moreover, the mean serum creatinine at baseline was 2.18 mg/dL, decreasing to 1.68 mg/dL after three months. This decline in serum creatinine levels suggests an improvement in kidney function during the course of the study. According to a study by Bhattacharya and colleagues [9], the mean serum creatinine level upon admission was 2.37 mg/dL. In contrast, after admission to the hospital, the mean serum creatinine levels increased to 2.96 mg/dL and 3.26 mg/dL after 24 and 48 hours, respectively. Additionally, 26.67% of the patients experienced oliguric renal failure. In another study by Lin et al., it was observed that the median serum creatinine level was 129.5 mmol/L, which was significantly higher compared to the present study. Additionally, the mean serum albumin value was 9.9 g/L, which was significantly lower than the findings of the present study [6].

The following observations were made on the basis of the distribution of patients according to AKI stage, 53.3% (n = 32) of the patients had AKIN stage l AKI. Out of these, 96.87% (n = 31) recovered, and only 3.12% (n = 1) progressed to CKD. Whereas 46.66% (n = 28) of the patients had AKIN stage 2 AKI. Among them, 82.14% (n = 23) recovered, and 17.85% (n = 5) progressed to CKD. None of the patients in the study population had AKIN stage 3 AKI. Upon evaluation, it was found that 95% (n = 57) of the patients had pre-renal AKI, while 5% (n = 3) had renal AKI due to sepsis. None of the patients had post-renal AKI. Among those with pre-renal AKI, all patients had hypoalbuminemia and were on diuretics, and 26.7% (n = 16) had a history of concomitant NSAID use. In another study conducted by Yaseen et al., which examined the short-term outcome of AKI in children, it was found that slightly over half of the children (65, 54.6%) made a full recovery. Among the 54 patients (45.4%) who did not recover, 41.2% (n = 49) developed CKD to varying degrees, and 4.2% (n = 5) passed away. Specifically, 14.3% (n = 17) advanced to CKD stage 2, 15.1% (n = 18) to CKD stage 3, 8.4% (n = 10) to CKD stage 4, and 3.4% (n = 4) to CKD stage 5/ESRD [5]. In the study by Bhattacharya and colleagues, it was found that the majority of patients (54.67%) had AKI stage 1, while 16.0% and 29.33% had AKI stages 2 and 3, respectively [9].

In a study conducted by Anigilaje and colleagues, the researchers investigated the causes of AKI. They found that the most prevalent factors leading to AKI were sepsis, accounting for 46.6% of cases, followed by acute glomerulonephritis (11.6%), diarrheal dehydration (11.6%), severe falciparum malaria (9.3%), and hemolytic-uremic syndrome (9.3%). This study focuses on the primary causes of AKI, highlighting the significance of sepsis as a leading contributor to this condition, along with other notable factors such as glomerulonephritis and various infectious diseases [10].

In the analysis of the histological class of the study population, the researchers made significant observations. FSGS was identified in 38.3% of the patients, indicating its prevalence as a histological finding. Minimal change disease (MCD) accounted for 31.7% of the cases, while membranous glomerulonephritis (MGN) was present in 21.7% of the patients. Additionally, 8.3% of the patients had a different histological diagnosis than MCD, FSGS, or MGN. Another study by Yassir et al. [11] revealed further insights into the underlying causes of NS within a larger context. Among the cases analyzed, membranous nephropathy (MN) emerged as the most prevalent cause, accounting for 22.2% of instances. The second most common cause was MCD, observed in 20.6% of patients. Other notable causes included lupus nephritis in 13.6% of patients, amyloidosis in 13.2% of patients, FSGS in 3.4% of patients, membranoproliferative glomerulonephritis (MPGN) in 8.2% of patients, diabetic nephropathy (DN) in 6.2% of patients, IgA nephropathy (IgAN) in 2.7% of patients and post-infectious glomerulonephritis (PIGN) in 1.9% of patients. Among patients with MCD, a remarkable 100% achieved complete remission, highlighting the favorable prognosis associated with this condition. For individuals diagnosed with FSGS, the outcomes varied, with 52.17% experiencing complete remission, 26.08% achieving partial remission, and 21.73% showing no response to treatment. Among the patients of MGN, all 13 patients (100%) achieved partial remission after three months, while complete remission was not observed. Among other causes of NS, including C3 glomerulonephritis and IgA nephropathy, a majority of patients (80%) displayed no response to treatment, while only one patient achieved partial remission. Only 3.3% (n = 2) of patients needed RRT.

Limitations

This cross-sectional design may have caused interference among the data, as no control population was enrolled in the study. Medications that may influence the clinical profile were not considered in the present study. The number of enrolled subjects was small, and this may have influenced the power of the statistical analyses. Larger prospective trials are needed to assess the clinical profile of patients with NS in AKI patients.

## Conclusions

The findings of the present study provide valuable insights into the characteristics and prognosis of this patient population, contributing to a better understanding of NS with AKI in adults. These findings indicate favorable changes in proteinuria, serum albumin, total cholesterol, and serum creatinine levels after three months of treatment. They suggest that the therapeutic intervention had a positive impact on the patients' renal function and overall clinical profile.
